# Gene expression association study in feline mammary carcinomas

**DOI:** 10.1371/journal.pone.0221776

**Published:** 2019-08-28

**Authors:** Daniela Ferreira, Bárbara Martins, Maria Soares, Jorge Correia, Filomena Adega, Fernando Ferreira, Raquel Chaves

**Affiliations:** 1 CAG - Laboratory of Cytogenomics and Animal Genomics, Department of Genetics and Biotechnology, University of Trás-os-Montes e Alto Douro, Vila Real, Portugal; 2 BioISI - Biosystems & Integrative Sciences Institute, Faculty of Sciences, University of Lisbon, Lisbon, Portugal; 3 CBiOS - Research Center for Biosciences & Health Technologies, Faculdade de Medicina Veterinária, Universidade Lusófona de Humanidades e Tecnologias, Lisbon, Portugal; 4 CIISA - Centro de Investigação Interdisciplinar em Sanidade Animal, Faculdade de Medicina Veterinária, Universidade de Lisboa, Avenida da Universidade Técnica, Lisboa, Portugal; Institut national de la recherche scientifique, CANADA

## Abstract

Works on cancer-related genes expression using feline mammary carcinomas (FMCs) are scarce but crucial, not only to validate these tumours as models for human breast cancer studies but also to improve small animal practice. Here, the expression of the cancer-related genes *TP53*, *CCND1*, *FUS*, *YBX1*, *PTBP1*, *c-MYC* and *PKM2* was evaluated by real-time RT-qPCR, in a population of FMCs clinically characterized and compared with the disease-free tissue of the same individual. In most of the FMCs analysed, RNA quantification revealed normal expression levels for *TP53*, *c-MYC*, *YBX1* and *FUS*, but overexpression in the genes *CCND1*, *PTBP1* and *PKM2*. The expression levels of these cancer-related genes are strongly correlated with each other, with exception of *c-MYC* and *PKM2* genes. The integration of clinicopathological data with the transcriptional levels revealed several associations. The oral contraceptive administration showed to be positively related with the *TP53*, *YBX1*, *CCND1*, *FUS* and *PTBP1* RNA levels. Positive associations were found between tumour size and *YBX1* RNA, and lymph node metastasis with *c-MYC* RNA levels. This work allowed to verify that many of these cancer-related genes are associated but may also, indirectly, influence other genes, creating a complex molecular cancer network that in the future can provide new cancer biomarkers.

## Introduction

Feline mammary carcinomas (FMC) have been emerging as valuable models for human breast cancer (HBC), allowing to uncover the mechanisms underlying tumorigenesis, to understand its origin/progression and to assist in the development of novel therapies [1]. The domestic cat is highly affected by spontaneous mammary tumours which are, in many aspects (e.g., clinicopathologically or histologically [2], among others) similar to HBC. Although the number of studies claiming the importance of FMC models is increasing, there is still a lack of consistency among them [1]. One of the drawbacks of this situation is the scarcity of association studies regarding cancer-related gene expression, which will allow to better characterize FMCs at the molecular level. Although several are the genes associated with HBC, in this study, we have chosen a specific set of cancer-related genes as such: *TP53*, *CCND1*, *FUS*, *YBX1*, *PTBP1*, *c-MYC* and *PKM2*. This cancer gene panel was selected based on the following assumptions: the information in FMCs about these genes is scarce or inexistent; these genes are conserved between cat and human; the function of these genes that is ascribed in HBC; and the pathways in which the products of these genes are involved, establishing a molecular cancer network that is important to analyse as a whole.

*TP53* is a tumour suppressor gene frequently mutated in human cancers [3, 4] but is still controversial its value as a prognostic marker in HBC [5]. P53 is a key player in cell cycle regulation and DNA damage response [3, 6] and its loss results in uncontrolled proliferation of damaged cells [6, 7]. Few mutations on *TP53* were reported in cat tumour tissues [1, 8], as well as, the accumulation of P53 protein in 35–45% of the FMCs already analysed [9, 10].

Cyclin D1 (coded by *CCND1* gene) is an oncoprotein overexpressed in about 50% HBCs and associated with cancer onset and progression [11, 12] due to its role in cell cycle initiation. Also, *CCND1* is amplified in 5–20% of HBCs and this occurs preferentially in ER positive tumours, being its prognostic significance proposed by different authors [13–15]. In cat, Murakami and collaborators evaluated the expression of Cyclin D1 protein in 37 feline mammary carcinomas and only 2 cases showed overexpression [9].

Fused in Sarcoma (FUS) is an RNA/DNA binding protein, being an important player in alternative splicing, transcription, DNA damage repair and stress response. Little is known about its contribution to cancer [16–18], but it is possible that this protein regulates the expression of many cancer-related genes, promoting tumorigenesis [19].

Y-box binding protein 1 (YBX1) is an oncoprotein that binds to the Y-box motif of gene promoters [20, 21], and its overexpression in HBC is related with more aggressive tumours, poor prognosis, relapse and drug resistance, indicating its potential as a prognostic biomarker [20, 22]. YBX1 has also been linked to the expression of other cancer-related genes (e.g., *c-MYC*, *CCND1*) [23–25].

Polypyrimidine tract-binding protein 1 (PTBP1) is an RNA-binding protein with functions at mRNA stability, transport, polyadenylation and splicing [26, 27]. This protein is overexpressed in different human cancers [27, 28], including breast cancer, promoting metastasis and cell proliferation [28].

c-MYC is a DNA-binding transcription factor that regulates numerous genes involved in critical biological processes [29, 30], being upregulated in several human cancer types, and associated with tumour aggressiveness and poor clinical outcome [31]. In HBC, *c-MYC* RNA expression is increased in 22–35% of the tumours analysed and protein expression is reported to be increased in up to 70% of all the cases studied [32–34]. Regarding FMCs, only one recent report stated that *c-MYC* gene was upregulated in 60% of the feline mammary adenocarcinomas analysed (in a small number of samples, n = 5) [35].

Pyruvate Kinase Muscle Isozyme (PKM2) is a moonlight protein (def., multifunctional protein that performs autonomous and often unrelated functions, without partitioning these functions into different domains of the protein [36]), acting as a pyruvate kinase at the cytoplasm and as a protein kinase at the cell nucleus. At the nucleus, PKM2 is a coactivator for the expression of several genes such as *CCND1* and *c-MYC* [37]. *PKM2* is also spliced by PTBP1, which in turn depends on c-MYC as its transcription factor [38–40]. In HBC, *PKM2* gene is frequently overexpressed (both at the protein and RNA levels) and associated with poor prognosis and overall survival and is involved in chemosensitivity to certain drugs [39, 41].

To our best knowledge, in FMCs no studies were performed to evaluate the expression of the following genes: *FUS*, *YBX1*, *PTBP1* and *PKM2*.

Bearing in mind the objective of contributing to deep knowledge on a panel of cancer-related genes (*TP53*, *CCND1*, *FUS*, *YBX1*, *PTBP1*, *c-MYC* and *PKM2*) in FMCs and its relation with clinicopathological parameters. We established an association study to disclose its RNA profiles (through absolute quantification by real-time RT-qPCR) in a group of FMCs, using the disease-free tissue (DFT) from each individual, as reference.

## Materials and methods

### Mammary tissues collection and characterization

The 27 mammary malignant tumours collected from female cats and the corresponding disease-free tissues were received from different veterinary hospitals and private practices, with the owner’s consent and in accordance with the EU Directive 2010/63/EU and the ethical approval was obtained in the frame of a project from the Science and Technology Foundation (FCT) of the Portuguese government with the reference PTDC/CVT-EPI/3638/2014. The tumours were histologically classified according to the World Health Organization (WHO) criteria for canine and feline mammary neoplasms and the Elston & Ellis (EE) grading system [42] and the Mills grading system (adapted for FMC) [43] were used to determine the malignancy grade. Cats from different breeds and age ranging from 7 to 17 years old were clinically evaluated, in particularly, the mammary glands and regional lymph nodes were physically inspected. The disease-free tissues were collected from another mammary gland and a histopathological confirmation of the absence of preneoplastic alterations was performed. The following clinicopathological parameters were recorded when possible: size of the tumour (T1 < 2 cm; T2 > 2 cm and < 3 cm; T3 > 3 cm), reproductive status, administration of oral contraception, mastectomy accompanied by ovariohysterectomy (OVH), presence of multiple tumours, lymph node metastasis, necrosis, lymphovascular invasion and lymphocytic inflammation and skin ulceration. Surgical excision of the tumours and normal mammary tissues was performed for all the animals and the tissues were immediately preserved in an RNA stabilization solution (RNA Later Tissue Collection, Ambion) and frozen at (−80°C) to prevent RNA degradation by RNases. A piece of the sample was formalin-fixed and paraffin embedded for the immunohistochemistry (IHC) analysis, being also collected a sample of blood of each animal for the serum analysis. Clinical staging was performed using the TNM system and animals were classified in four stages [44]. All the animals were followed up after the tumours removal for the survival, recurrence and type of recurrence. The IHC detection of the proteins HER2 (Human Epidermal growth factor Receptor 2, classified as positive when 3+, equivocal 2+ and negative 1+ or 0), Ki-67 (that is a proliferation marker protein, considered low when <14% and high ≥14%), PR (Progesterone Receptor, evaluated as negative when <3 and positive when ≥3), ER (Estrogen Receptor, considered as negative when <3 and positive when ≥3) and CK5/6 (Cytokeratin 5/6, positive when >1% of cells were immunoreactive) and its quantification analysis in the mammary tumours were performed according to the method described in Soares et al. [45]. The analysis of these five proteins allowed us to obtain a molecular classification of the tumours, applying the St. Gallen International Expert Consensus panel [2, 46].

### Genomic DNA and RNA extraction

RNA was isolated with the mirVana^™^ miRNA Isolation Kit (Ambion, Life Technologies) as described by the manufacturer and thereafter submitted to DNA degradation with the TURBO DNA-free Kit (Ambion, Life Technologies).

### RNA expression analysis by real-time RT-qPCR

For *TP53*, *CCND1*, *FUS*, *YBX1*, *PTBP1*, *c-MYC* and *PKM2* RNA quantification (primers in S1 Table), was used the standard curve method described in Chaves et al. [47] (standard curve parameters in S2 Table). For the expression quantification, it was used 80 ng of RNA and the Verso 1-Step RT-qPCR kit, SYBR Green, ROX (Thermo Scientific) following the recommendations of the manufacturer. The reactions were carried out in a 48-well optical plate (StepOne real-time PCR system, Applied Biosystems, Thermo Fisher Scientific) at 50 °C for 15 min and 95 °C for 15 min, followed by 40 cycles of 95 °C for 15 sec and 60°C for 1 min. Subsequently, a melt curve was performed to evaluate the primers specificity. All reactions were performed in triplicate, and negative controls (without RNA and without Reverse Transcriptase enzyme) were also included in the plate. The data were analysed using the same parameters and the StepOne software (version 2.2.2, Applied Biosystems, Thermo Fisher Scientific).

### Statistical analysis

The statistical software SPSS (Statistical Package for the Social Sciences, version 17.0), the GraphPad Prism 6 (version 6.01) and the R software (The R Foundation for Statistical Computing, 3.3.1 version) were used for the statistical analysis. The Student’s t-test (two-tailed) was applied for the analysis of the real-time RT-qPCR results. Statistical associations among the clinicopathological parameters and the RNA data were evaluated using the ANOVA test (for analysing continuous variables with categorical variables). The Pearson’s correlation test was performed in order to verify the correlation between continuous variables. As the RNA quantification data did not present a Gaussian distribution, the values were transformed with the log function in order to normalize the its distribution. The correlogram was made with GraphPad Prism 6 (version 6.01) and R software’s (The R Foundation for Statistical Computing, 3.3.1 version). The correlogram representation is the output of the R software but r-values were corrected by the ones from GraphPad software (some analysis presented a different “n”). All values are expressed as mean ± SD (standard deviation). The exceptions are the data presented in the box-plot graphics that represents the median, quartiles, and extreme values within a category. In all statistical comparisons, *p*< 0.05 was established as representing significant difference.

## Results

### Gene expression profiling in feline mammary carcinomas

**Fig 1:**
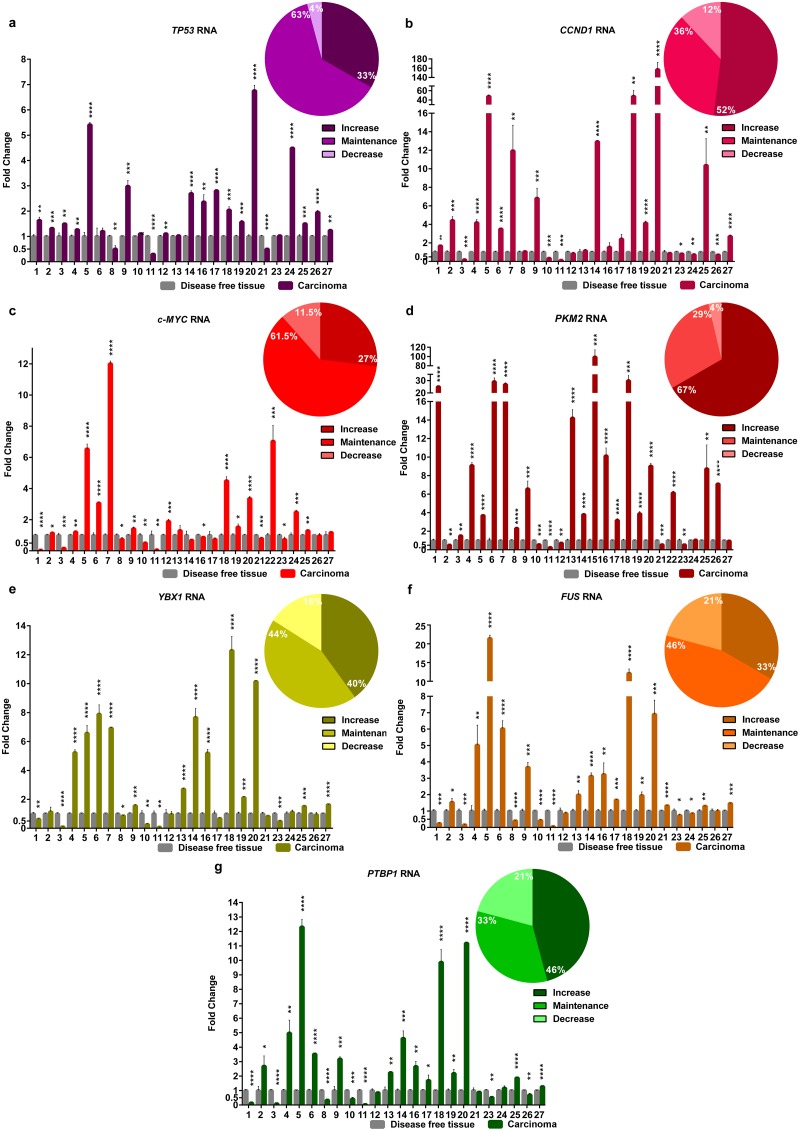
Profiling the RNA levels of cancer-related genes in the analysed FMCs. Fold change of *TP53* (**a**), *CCND1* (**b**), *c-MYC* (**c**), *PKM2* (**d**), *YBX1* (**e**), *FUS* (**f**) and *PTBP1* (**g**) RNAs in FMC, evaluated by real-time RT-qPCR and using a DFT (disease-free tissue) sample of the same individual as reference. Each quantification graphic also presents the percentage of tumours with increase (≥2-folds), maintenance (between 0.5 and 2-folds) or decrease (≤0.5-folds) RNA levels of each gene. Values are mean ± SD of three replicates. *p≤0.05, **p≤0.01, ***p≤0.001, ****p≤0.0001 was determined by Student’s t-test.

A great number of cancer-related genes expression remains to be properly characterized in FMCs. In this work, we have quantified the expression (RNA) of several cancer-related genes in a set of FMCs and in the DFT from the same individual (used as reference), by real-time RT-qPCR. An overexpressed gene was considered when the FMC presents an increase of ≥2-folds, a decreased in the gene expression corresponds to values of ≤0.50-fold and finally a maintained gene expression present values between 0.5 and 2-folds. All this analysis is always based in comparison with the respective DFT. In most of the FMCs, our analysis revealed that: the expression of *TP53* is maintained in 63% (15/24) and overexpressed in 33% (8/24) (Fig 1a and S3 Table); *CCND1* gene is overexpressed in 52% (14/27) (Fig 1b and S4 Table); the expression of *c-MYC* gene is maintained in 61.5% (16/26) and increased in 27% (7/26) (Fig 1c and S5 Table); *PKM2* is overexpressed in 67% (18/27) (Fig 1d and S6 Table); the expression levels of *YBX1* is maintained in 44% (11/25), being the number of cases that presented overexpression similar (10/25, 40%) (Fig 1e and S7 Table); *FUS* gene expression levels is maintained in 46% (11/24) with 33% of FMCs showing increased expression (8/24) (Fig 1f and S8 Table); and, finally, the gene expression of *PTBP1* is increased in 46% (11/24) (Fig 1g and S9 Table). In all the FMCs analysed and for the gene panel used, only a small number of FMCs presented a decreased expression.

**Fig 2:**
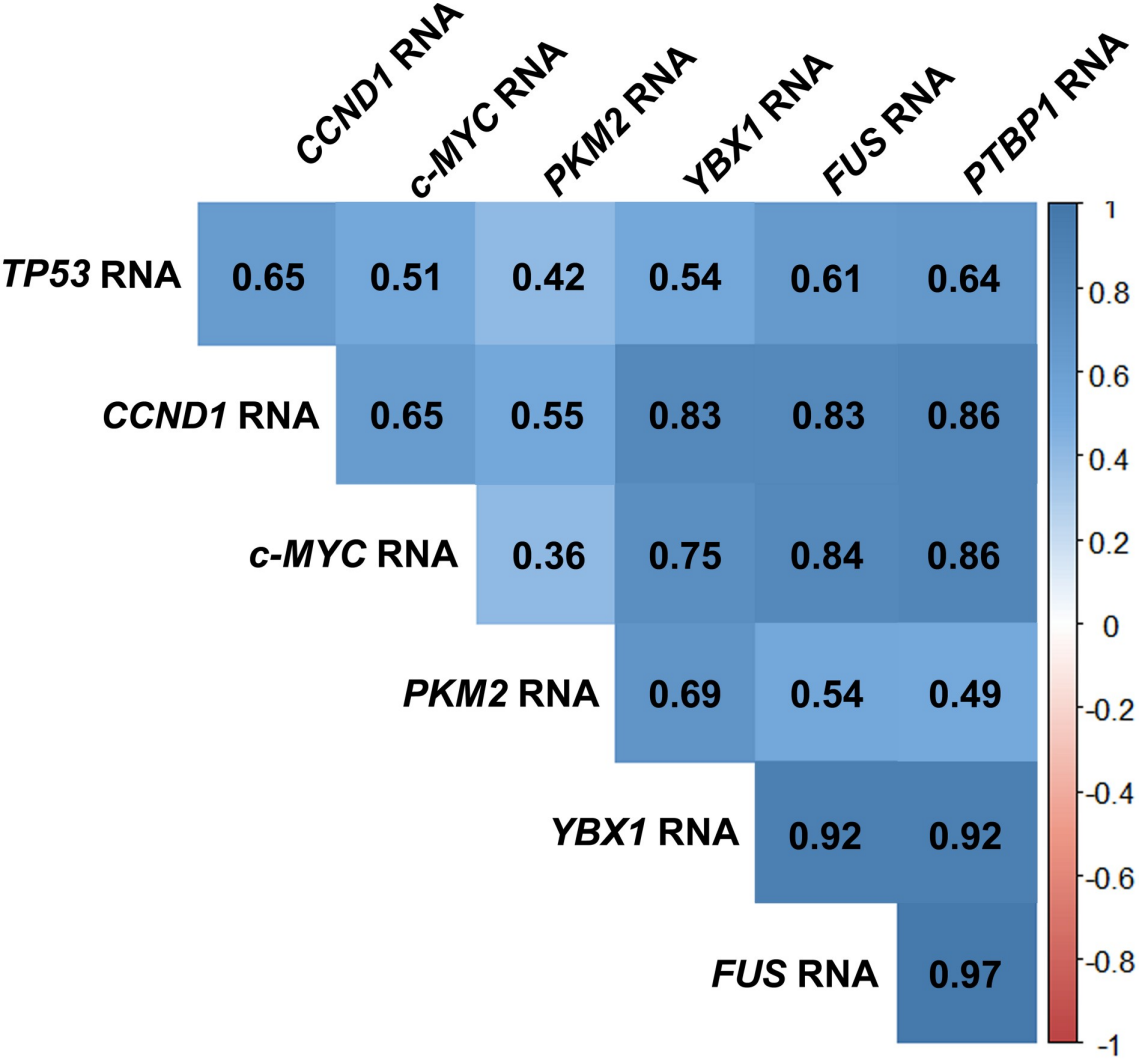
Correlation analysis of the RNA expression levels of *TP53*, *CCND1*, *c-MYC*, *PKM2*, *YBX1*, *FUS* and *PTBP1*. This correlogram was obtained using the R software.

Also, the analysis between the RNA quantification data of all the genes under study allowed us to verify that all the expression levels in the FMCs are correlated in a statistically significant fashion (with the r-value ranging between 0.42 and 0.97, the p-value between 0.044 and >0.0001, n = 24 or 25) with exception of *c-MYC* and *PKM2* (r = 0.36, p = 0.073, n = 26) (Fig 2).

### Cancer-related genes expression association with clinicopathological parameters

**Fig 3:**
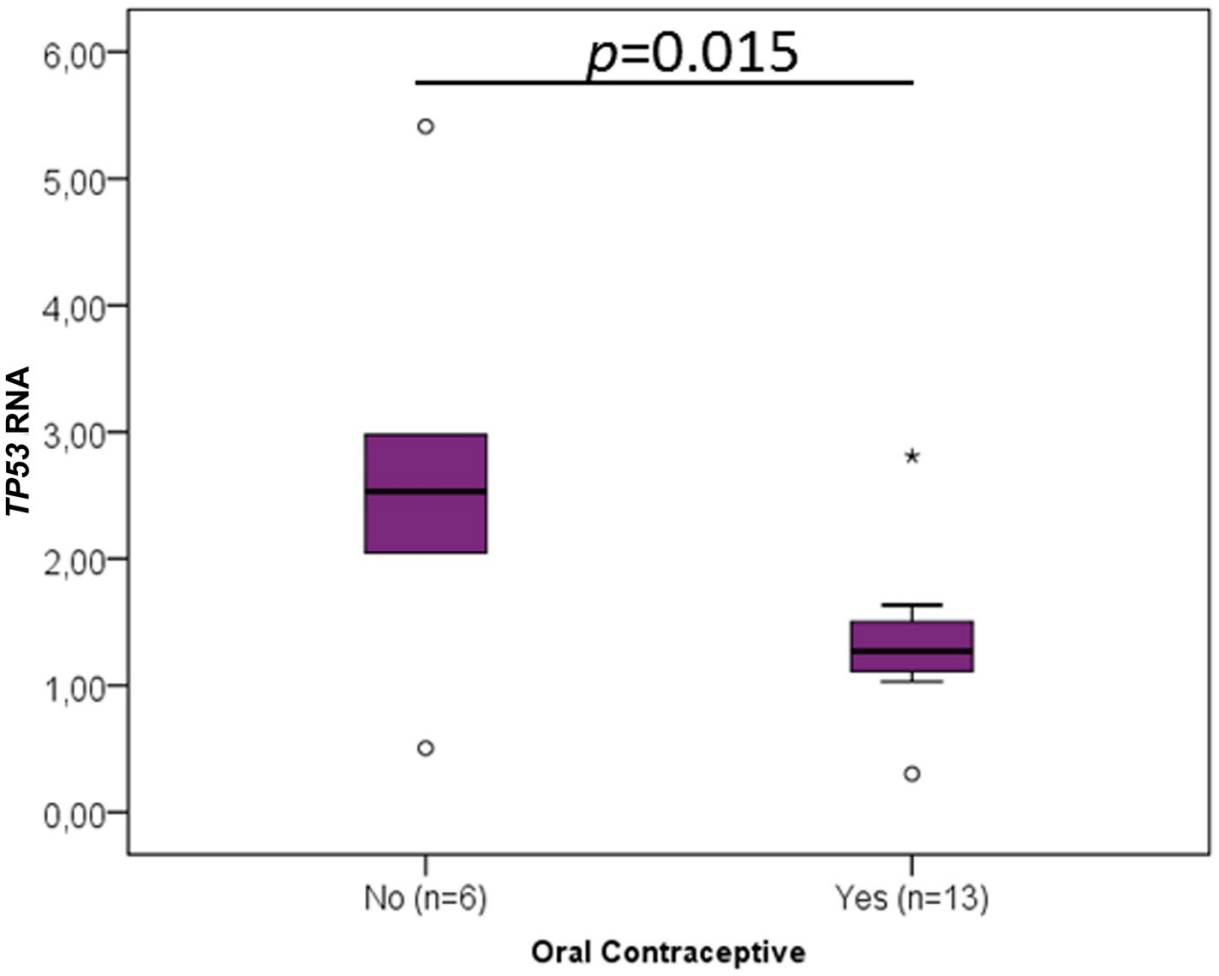
*TP53* RNA association with clinicopathological parameters. Box plot graphical representation of the analysis of *TP53* RNA with oral contraceptive administration. The data are presented as box-plot graphic that represents the median, quartiles, and extreme values within a category. The *p*-value is presented and obtained by using the one-way ANOVA test.

**Fig 4:**
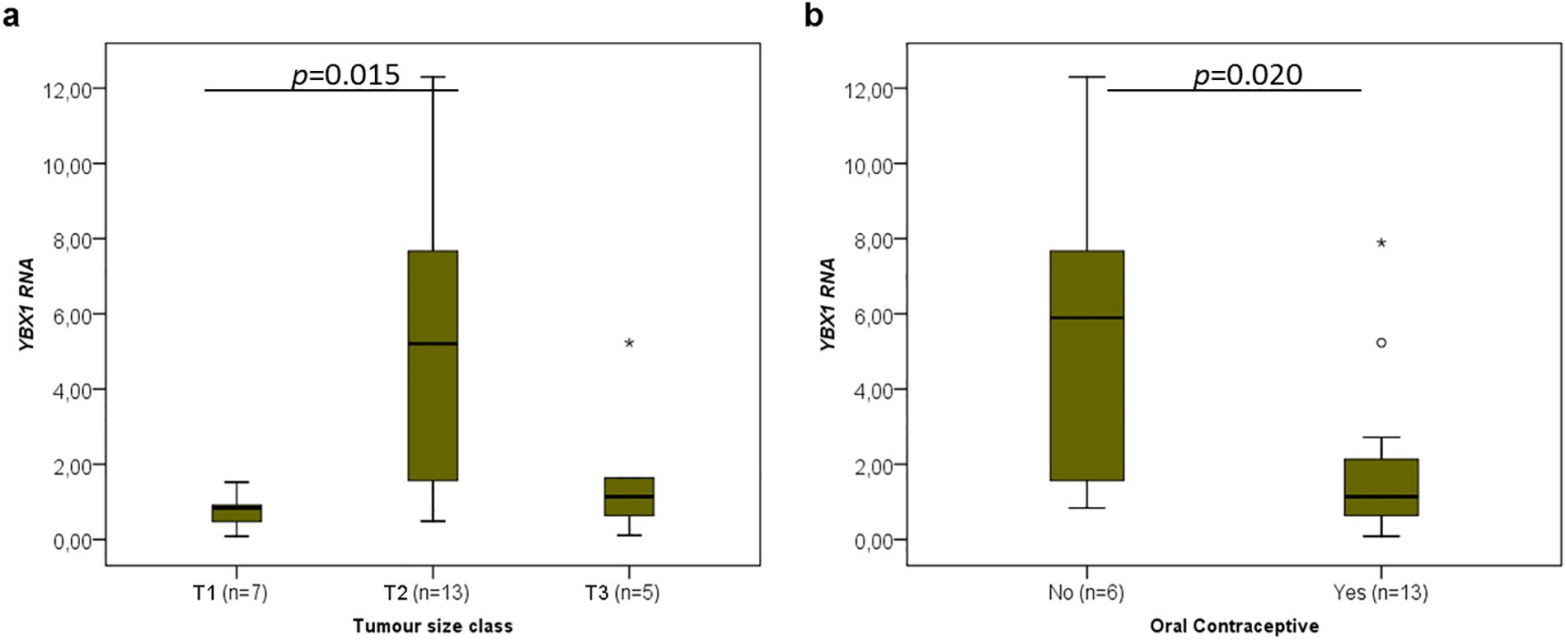
*YBX1* RNA association with clinicopathological parameters. Box plot graphical representation of the analysis of *YBX1* RNA levels with tumour size classes (a) and oral contraceptive administration (b). The *p*-value is presented in each graphic and obtained by using the one-way ANOVA test (Tukey Post Hoc Multiple Comparisons).

**Fig 5:**
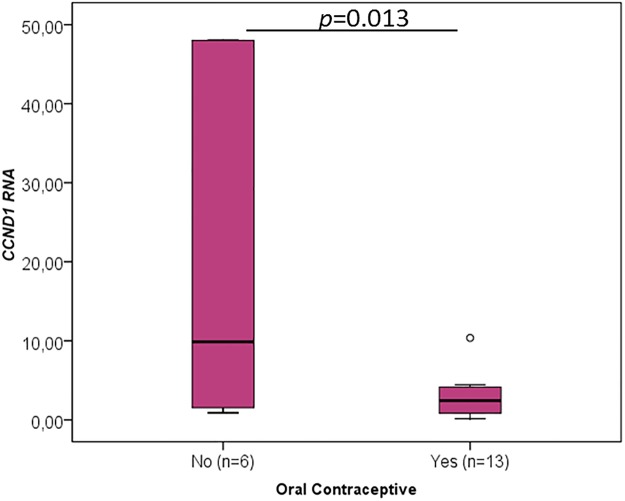
*CCND1* RNA association with clinicopathological parameters. Box plot graphical representation of the analysis of *CCND1* RNA with oral contraceptive administration. The *p*-value is presented and obtained by using the one-way ANOVA test.

**Fig 6:**
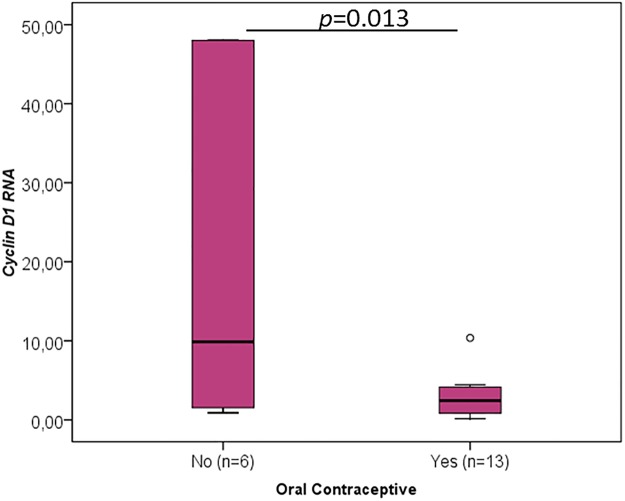
*FUS* RNA association with clinicopathological parameters. Box plot graphical representation of the analysis of *FUS* RNA with oral contraceptive administration. The *p*-value is presented and obtained by using the one-way ANOVA test.

**Fig 7:**
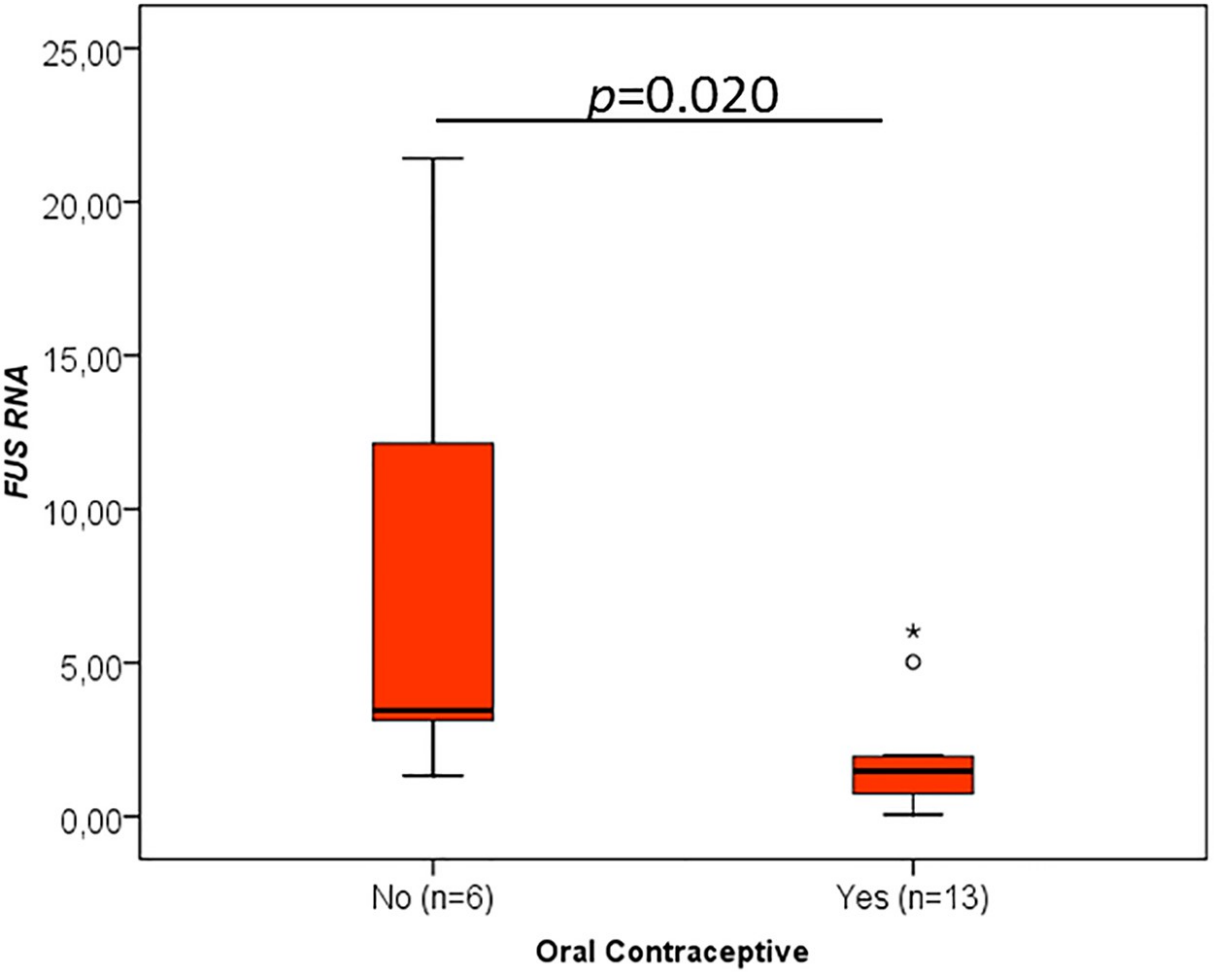
*PTBP1* RNA association with clinicopathological parameters. Box plot graphical representation of the analysis of *PTBP1* RNA with oral contraceptive administration. The *p*-value is presented and obtained by using the one-way ANOVA test.

**Fig 8:**
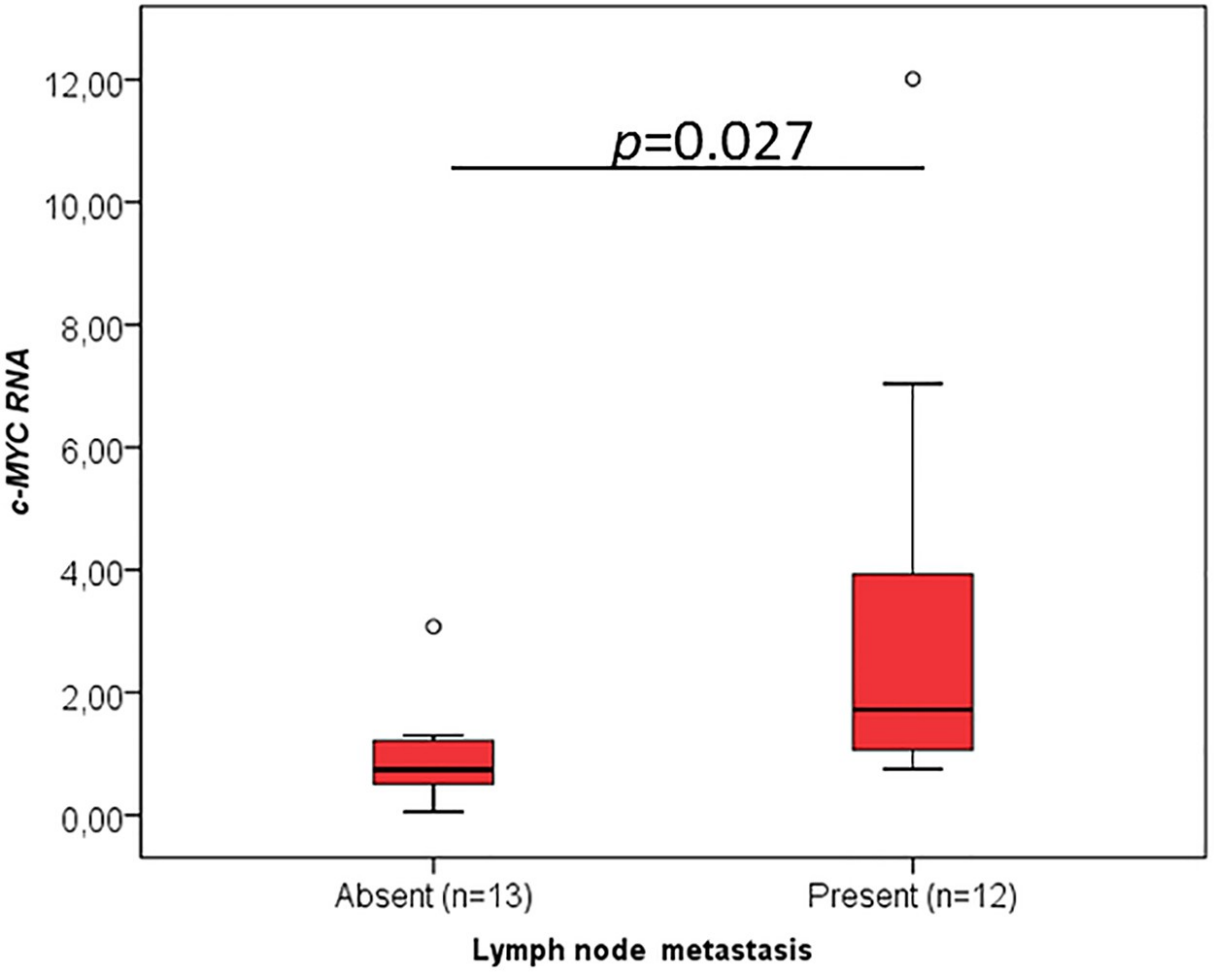
*c-MYC* RNA association with clinicopathological parameters. Box plot graphical representation of the analysis of *c-MYC* RNA with Lymph node metastasis. The *p*-value is presented and obtained by using the one-way ANOVA test (Tukey Post Hoc Multiple Comparisons).

When the different clinicopathological data were analysed concerning the RNA levels of the cancer-critical genes, an interesting association was found between the oral contraceptive administration and RNA levels of *TP53* (p = 0.015, Fig 3, Table 1), *YBX1* (p = 0.020, Fig 4b, Table 2), *CCND1* (p = 0.013, Fig 5, Table 3), *FUS* (p = 0.020, Fig 6, Table 4) and *PTBP1* (p = 0.010, Fig 7, Table 5). In fact, the expression levels of all these genes are inferior in animals’ subjected to oral contraceptive administration. The association between oral contraception administration (compared to animals which were never exposed to oral contraceptives) and the expression of these cancer-related genes has not yet been reported in cats. Regarding tumour size, *YBX1* expression was significantly higher in T2 (2–3 cm) tumours than in T1 (<2 cm) tumours (p = 0.012, Fig 4a, Table 2). The tumours with more than 3 cm (classified as T3) didn’t present an association with *YBX1* RNA levels. *TP53* RNA levels also demonstrated an association with tumour size (in the one-way ANOVA, Table 1) but the Post-Hoc tests are not statistically significant. Regarding *c-MYC*, a positive association with the lymph node metastasis (p = 0.027, n = 25) (Fig 8, Table 6) was found; that is, the levels of *c-MYC* RNA are higher in cats with the tumours and lymph node metastasis. Even if it was observed a positive association of *c-MYC* RNA levels with skin ulceration (p<0.0001, n = 26), a higher number of animals is required for further validation. *PKM2* RNA levels demonstrated to be associated with the malignancy grade by EE grading system [42] (p = 0.008, n = 27) (Table 7). The cases with malignancy grade I are those that presented the highest *PKM2* expression levels. However, cases with malignancy grade II demonstrated the lowest expression of *PKM2*. Nevertheless, when the FMCs are classified concerning the malignancy grade by the Mills grading system (published for FMCs [43]), its association with *PKM2* RNA levels is not statistically significant. *PKM2* RNA levels also demonstrated to be related with the molecular classification (p<0.001, n = 27) (Table 7). The subtypes LA (luminal A) and LB (Luminal B) presented higher *PKM2* expression, whereas the TN (triple negative) subtype had the lowest levels. Nevertheless, the malignancy grade I (by EE grading system) and LA tumours are underrepresented in our sample set (FMC are often highly aggressive). In the future, will be important to increase the number of tumours with these features to obtain more robust results. Although survival data and prognostic analyses were taken into consideration in our evaluation, no statistically significant results were achieved, and for that reason, these data are not shown.

**Table 1:** *TP53* RNA relation with clinicopathological parameters. This analysis was performed using one-way ANOVA.

Clinicopathological parameter		*TP53 RNA* *Mean*	*P*	*Clinicopathological parameter*		*TP53 RNA* *Mean*	*p*
*Tumour size*	T1 (< 2 cm)	1.12	0.041* (n = 24)	*Lymphovascular invasion*	Present	2.70	0.280 (n = 24)
	T2 (2–3 cm)	2.80					
					Absent	1.83	
	T3 (> 3 cm)	1.39					
*Ck5/6 index*	High	2.22	0.471 (n = 24)	*Lymphocytic inflammation*	Present	2.26	0.285 (n = 24)
	Low	1.73			Absent	1.52	
*Sterilized*	Yes	2.13	0.701 (n = 24)	*Ki-67 index*	High	2.17	0.284 (n = 24)
	No	1.88			Low	1.23	
*Oral contraceptive*	Yes	1.35	0.015* (n = 19)	*PR status*	Positive	2.19	0.445 (n = 24)
	No	2.67			Negative	1.66	
*OVH with mastectomy*	Yes	1.95	0.799 (n = 11)	*ER status*	Positive	2.01	0.993 (n = 24)
	No	1.57			Negative	2.02	
*Multiple tumours*	No	1.64	0.598 (n = 24)	*HER2 status*	Positive	1.66	0.350 (n = 24)
	Multicentric	2.20			Equivocal	2.68	
	Multicentric/multifocal	2.50			Negative	1.68	
*Lymph node metastasis*	Present	2.51	0.055 (n = 23)	*Molecular classification*	LB	1.73	0.818 (n = 24)
	Absent	1.37			HER2	2.35	
					LBHER2	2.54	
*Tumour stage*	1	1.25	0.486 (n = 24)		LA	-	
	2	2.28			TN normal	1.03	
	3	2.20			TN basal	1.73	
*EE grading Malignancy grade*	I	0.50	0.584 (n = 24)	*Necrosis*	Present	2.34	0.111 (n = 24)
	II	1.72			Absent	1.22	
			
	III	2.13					
*Mills grading Malignancy grade*	I		0.387 (n = 24)				
	II	2.22					
	III	1.61					

* Indicates p≤0.05.

OVH–ovariohysterectomy.

**Table 2:** *YBX1* RNA association with clinicopathological parameters. This analysis was performed using one-way ANOVA test.

Clinicopathological parameter		*YBX1 RNA* *Mean*	*P*	*Clinicopathological parameter*		*YBX1 RNA* *Mean*	*p*
*Tumour size*	T1 (< 2 cm)	0.75	0.012* (n = 25)	*Lymphovascular invasion*	Present	3.75	0.697 (n = 25)
	T2 (2–3 cm)	5.05					
					Absent	3.05	
	T3 (> 3 cm)	1.75					
*Skin ulceration*	Present	6.92	0.285 (n = 25)	*Lymphocytic inflammation*	Present	3.57	0.440 (n = 25)
	Absent	3.03			Absent	2.38	
*Sterilized*	Yes	3.17	0.837 (n = 24)	*Ki-67 index*	High	3.50	0.375 (n = 25)
	No	2.87			Low	1.92	
*Oral contraceptive*	Yes	1.94	0.020* (n = 19)	*PR status*	Positive	3.72	0.320 (n = 25)
	No	5.69			Negative	2.24	
*OVH with mastectomy*	Yes	3.42	0.267 (n = 11)	*ER status*	Positive	3.19	0.997 (n = 25)
	No	0.37			Negative	3.19	
*Multiple tumours*	No	3.55	0.919 (n = 25)	*HER2 status*	Positive	0.76	0.605 (n = 25)
	Multicentric	3.00			Equivocal	3.25	
	Multicentric/multifocal	2.81			Negative	3.49	
*Lymph node metastasis*	Present	3.94	0.260 (n = 24)	*Molecular classification*	LB	4.18	0.725 (n = 25)
	Absent	2.29			HER2	2.60	
					LBHER2	2.87	
*Tumour stage*	1	0.68	0.130 (n = 25)		LA	-	
	2	5.07			TN normal	0.49	
	3	3.40			TN basal	1.23	
*EE grading Malignancy grade*	I	0.83	0.421 (n = 25)	*Necrosis*	Present	3.50	0.485 (n = 25)
	II	1.10			Absent	2.38	
	III	3.60					
*Mills grading Malignancy grade*	I		0.271 (n = 25)	*Ck5/6 index*	High	3.89	0.268 (n = 25)
	II	2.65					
	III	4.33			Low	2.30	

* Indicates p*≤0*.*05*

OVH–ovariohysterectomy.

**Table 3:** *CCND1* RNA relation with clinicopathological parameters. This analysis was performed using one-way ANOVA test.

Clinicopathological parameter		*CCND1 RNA* *Mean*	*P*	*Clinicopathological parameter*		*CCND1 RNA* *Mean*	*p*
*Tumour size*	T1 (< 2 cm)	2.30	0.306 (n = 25)	*Lymphovascular invasion*	Present	33.49	0.120 (n = 25)
	T2 (2–3 cm)	22.89					
					Absent	7.97	
	T3 (> 3 cm)	2.64					
*Skin ulceration*	Present	11.95	0.973 (n = 25)	*Lymphocytic inflammation*	Present	17.16	0.373 (n = 25)
	Absent	13.12			Absent	4.40	
*Sterilized*	Yes	9.64	0.589 (n = 24)	*Ki-67 index*	High	15.51	0.468 (n = 25)
	No	17.24			Low	3.34	
*Oral contraceptive*	Yes	2.82	0.013* (n = 19)	*PR status*	Positive	15.43	0.641 (n = 25)
	No	19.71			Negative	8.89	
*OVH with mastectomy*	Yes	20.86	0.611 (n = 11)	*ER status*	Positive	5.01	0.499 (n = 25)
	No	0.95			Negative	15.62	
*Multiple tumours*	No	10.69	0.742 (n = 25)	*HER2 status*	Positive	1.66	0.379 (n = 25)
	Multicentric	18.44			Equivocal	25.33	
	Multicentric/multifocal	4.26			Negative	6.83	
*Lymph node metastasis*	Present	21.58	0.172 (n = 19)	*Molecular classification*	LB	7.60	0.890 (n = 25)
	Absent	3.19			HER2	17.53	
					LBHER2	22.34	
*Tumour stage*	1	2.84	0.740 (n = 25)		LA	-	
	2	13.29			TN normal	0.82	
	3	16.41			TN basal	5.54	
*EE grading Malignancy grade*	I	0.89	0.765 (n = 25)	*Necrosis*	Present	15.07	0.635 (n = 25)
	II	1.99					
					Absent	7.95	
	III	15.24					
*Mills grading Malignancy grade*	I		0.638 (n = 25)	*Ck5/6 index*	High	18.07	0.399 (n = 25)
	II	15.24			Low	6.71	
	III	8.47					

* *Indicates* p*≤0*.*05*

OVH–ovariohysterectomy.

**Table 4:** *FUS* RNA relation with clinicopathological parameters. This analysis was performed using one-way ANOVA.

*Clinicopathological parameter*		*FUS RNA* *Mean*	*P*	*Clinicopathological parameter*		*FUS RNA* *Mean*	*p*
*Tumour size*	T1 (< 2 cm)	0.87	0.106 (n = 24)	*Lymphovascular invasion*	Present	2.76	0.809 (n = 24)
	T2 (2–3 cm)	5.25					
					Absent	3.35	
	T3 (> 3 cm)	1.69					
*Ck5/6 index*	High	3.10	0.882 (n = 24)	*Lymphocytic inflammation*	Present	3.88	0.359 (n = 24)
	Low	3.41			Absent	1.94	
*Sterilized*	Yes	4.37	0.208 (n = 24)	*Ki-67 index*	High	3.68	0.306 (n = 24)
	No	1.88			Low	0.96	
*Oral contraceptive*	Yes	1.77	0.020* (n = 19)	*PR status*	Positive	3.04	0.792 (n = 24)
	No	7.49			Negative	3.61	
*OVH with mastectomy*	Yes	2.25	0.202 (n = 11)	*ER status*	Positive	1.53	0.379 (n = 24)
	No	0.21			Negative	3.68	
*Multiple tumours*	No	4.76	0.427 (n = 24)	*HER2 status*	Positive	1.50	0.740 (n = 24)
	Multicentric	2.01			Equivocal	4.19	
	Multicentric/multifocal	2.46			Negative	2.93	
*Lymph node metastasis*	Present	3.25	0.234 (n = 23)	*Molecular classification*	LB	3.45	0.460 (n = 24)
	Absent	1.81			HER2	6.67	
					LBHER2	1.94	
*Tumour stage*	1	0.96	0.147 (n = 24)		LA	-	
	2	6.66			TN normal	0.74	
	3	2.82			TN basal	1.15	
*EE grading Malignancy grade*	I	1.33	0.701 (n = 24)	*Necrosis*	Present	3.53	0.641 (n = 24)
	II	1.33			Absent	2.50	
	III	3.61					
*Mills grading Malignancy grade*	I		0.693 (n = 24)				
	II	2.95					
	III	3.79					

* *Indicates* p*≤0*.*05*

OVH–ovariohysterectomy.

**Table 5:** *PTBP1* RNA relation with clinicopathological parameters. This analysis was performed using one-way ANOVA.

*Clinicopathological parameter*		*PTBP1 RNA* *Mean*	*P*	*Clinicopathological parameter*		*PTBP1 RNA* *Mean*	*p*
*Tumour size*	T1 (< 2 cm)	0.88	0.059 (n = 24)	*Lymphovascular invasion*	Present	3.76	0.542 (n = 24)
	T2 (2–3 cm)	4.52					
					Absent	2.67	
	T3 (> 3 cm)	1.84					
*Ck5/6 index*	High	3.27	0.541 (n = 24)	*Lymphocytic inflammation*	Present	3.30	0.433 (n = 24)
	Low	2.37			Absent	2.09	
*Sterilized*	Yes	3.36	0.494 (n = 24)	*Ki-67 index*	High	3.31	0.201 (n = 24)
	No	2.35			Low	0.84	
*Oral contraceptive*	Yes	1.70	0.010* (n = 19)	*PR status*	Positive	3.07	0.731 (n = 24)
	No	5.59			Negative	2.54	
*OVH with mastectomy*	Yes	2.85	0.304 (n = 11)	*ER status*	Positive	1.76	0.424 (n = 24)
	No	0.12			Negative	3.19	
*Multiple tumours*	No	3.51	0.755 (n = 24)	*HER2 status*	Positive	1.29	0.611 (n = 24)
	Multicentric	2.61			Equivocal	3.80	
	Multicentric/multifocal	2.07			Negative	2.61	
*Lymph node metastasis*	Present	3.49	0.152 (n = 23)	*Molecular classification*	LB	3.04	0.754 (n = 24)
	Absent	1.72			HER2	5.01	
					LBHER2	2.56	
*Tumour stage*	1	0.99	0.259 (n = 24)		LA	-	
	2	4.64			TN normal	0.53	
	3	2.95			TN basal	1.29	
*EE grading Malignancy grade*	I	0.89	0.575 (n = 24)	*Necrosis*	Present	3.23	0.472 (n = 24)
	II	1.28			Absent	2.08	
	III	3.24					
*Mills grading Malignancy grade*	I		0.718 (n = 24)				
	II	2.71					
	III	3.27					

* *Indicates* p*≤0*.*05*

OVH–ovariohysterectomy.

**Table 6:** *c-MYC* RNA relation with clinicopathological parameters. This analysis was performed using one-way ANOVA test.

*Clinicopathological parameter*		*c-MYC RNA* *Mean*	*P*	*Clinicopathological parameter*		*c-MYC RNA* *Mean*	*p*
*Tumour size*	T1 (< 2 cm)	0.87	0.218 (n = 26)	*Lymphovascular invasion*	Present	1.42	0.501 (n = 26)
	T2 (2–3 cm)	3.04					
					Absent	2.35	
	T3 (> 3 cm)	1.80					
*Skin ulceration*	Present	9.53	<0.001* (n = 26)	*Lymphocytic inflammation*	Present	2.66	0.170 (n = 26)
	Absent	1.56			Absent	1.07	
*Sterilized*	Yes	1.97	0.588 (n = 25)	*Ki-67 index*	High	1.98	0.476 (n = 26)
	No	1.56			Low	2.96	
*Oral contraceptive*	Yes	1.53	0.345 (n = 20)	*PR status*	Positive	1.54	0.134 (n = 26)
	No	2.47			Negative	3.18	
*OVH with mastectomy*	Yes	1.27	0.404 (n = 12)	*ER status*	Positive	3.18	0.305 (n = 26)
	No	2.42			Negative	1.87	
*Multiple tumours*	No	1.91	0.791 (n = 26)	*HER2 status*	Positive	0.77	0.367 (n = 26)
	Multicentric	2.06			Equivocal	3.16	
	Multicentric/multifocal	2.93			Negative	1.76	
*Lymph node metastasis*	Present	3.17	0.027* (n = 25)	*Molecular classification*	LB	1.49	0.798 (n = 26)
	Absent	0.91			HER2	2.59	
					LBHER2	2.78	
*Tumour stage*	1	0.68	0.406 (n = 26)		LA	-	
	2	2.47			TN normal	0.73	
	3	2.54			TN basal	3.10	
*EE grading Malignancy grade*	I	0.80	0.725 (n = 26)	*Necrosis*	Present	2.52	0.286 (n = 26)
	II	1.27					
					Absent	1.22	
	III	2.35					
*Mills grading Malignancy grade*	I		0.989 (n = 26)	*Ck5/6 index*	High	1.96	0.650 (n = 26)
	II	2.16					
	III	2.18			Low	2.46	

* *Indicates* p*≤0*.*05*

OVH–ovariohysterectomy.

**Table 7:** *PKM2* RNA relation with clinicopathological parameters. This analysis was performed using one-way ANOVA.

*Clinicopathological parameter*		*PKM2 RNA* *Mean*	*P*	*Clinicopathological parameter*		*PKM2 RNA* *Mean*	*p*
*Tumour size*	T1 (< 2 cm)	2.34	0.222 (n = 27)	*Lymphovascular invasion*	Present	5.02	0.444 (n = 27)
	T2 (2–3 cm)	17.51			Absent	12.72	
	T3 (> 3 cm)	7.22					
*Skin ulceration*	Present	16.54	0.705 (n = 27)	*Lymphocytic inflammation*	Present	9.90	0.615 (n = 27)
	Absent	10.87			Absent	14.08	
*Sterilized*	Yes	14.05	0.364 (n = 26)	*Ki-67 index*	High	8.20	0.132 (n = 27)
	No	6.77			Low	22.11	
*Oral contraceptive*	Yes	13.54	0.688 (n = 21)	*PR status*	Positive	9.40	0.559 (n = 27)
	No	9.12			Negative	14.05	
*OVH with mastectomy*	Yes	5.39	0.281 (n = 12)	*ER status*	Positive	19.02	0.238 (n = 27)
	No	10.91			Negative	8.59	
*Multiple tumours*	No	7.18	0.180 (n = 27)	*HER2 status*	Positive	1.88	0.0.367 (n = 27)
	Multicentric	7.79			Equivocal	5.37	
	Multicentric/multifocal	24.55			Negative	15.80	
*Lymph node metastasis*	Present	8.70	0.511 (n = 26)	*Molecular classification*	LB	11.93	<0.001* (n = 27)
	Absent	14.05			HER2	1.50	
					LBHER2	5.95	
*Tumour stage*	1	2.67	0.127 (n = 27)		LA	99.04	
	2	25.08			TN normal	0.55	
	3	8.82			TN basal	7.33	
*EE grading Malignancy grade*	I	49.80	0.008* (n = 27)	*Necrosis*	Present	13.35	0.371 (n = 27)
	II	1.63					
					Absent	5.40	
	III	9.11					
*Mills grading Malignancy grade*	I		0.782 (n = 27)	*Ck5/6 index*	High	15.51	0.187 (n = 27)
	II	10.52					
	III	12.83			Low	5.16	

* *Indicates* p*≤0*.*05*

OVH–ovariohysterectomy.

## Discussion

FMCs have emerged as good models for HBC studies, besides its importance in fundamental research such as the discovery of cancer-related genes and its cellular pathways, and development of new treatments [1]. However, studies on the characterization of cancer-related genes expression in FMCs are still scarce. In this work, we analysed the expression of seven genes (*TP53*, *CCND1*, *FUS*, *YBX1*, *PTBP1*, *c-MYC* and *PKM2*) in 27 FMCs using disease-free tissue (from the same individual) as reference. Using this approach, we were able to overcome the genetic background variations among individuals, making the present analysis more accurate in identifying the alterations involved in these tumours [48, 49].

Most of the FMCs analysed maintained the RNA levels of *TP53* (63%), *c-MYC* (61.5%), *YBX1* (44%) and *FUS* (46%) when compared with the DFTs. These same genes are overexpressed in 33%, 27%, 40% and 33% respectively, of the FMCs analysed. In this study, the proportion of tumours presenting an upregulation of *TP53* (33%) is similar to the reported in a similar work in FMCs [50]. With regard to *c-MYC*, its overexpression in 27% of the FMCs analysed is consistent with the report, that refers an overexpression of this gene in 22–35% of HBC [32], contrasting to what have been reported in FMCs, where it appears to be upregulated (60%) (but in a small set of samples analysed) [35]. Also, the percentage of tumours that present *YBX1* upregulated is consistent with the data found for its protein in HBC [22, 51]. Regarding the other RNAs analysed, they revealed to be upregulated in most of the tumours, namely *CCND1* (52%), *PKM2* (67%) and *PTBP1* (46%). Indeed, in our study, the expression levels of *CCND1* RNA are in agreement with the ones presented for the respective protein levels in HBC [12], where the expression levels of *CCND1* RNA and protein showed a good correlation [52]. In parallel, the upregulation scenario of *PKM2* RNA found in the FMCs analysed is similar to that reported for the PKM2 protein in HBC [41, 53].

When the expression levels of these genes in the different FMCs samples was evaluated, a strong positive correlation was observed between almost all the cancer-related genes under study (except for *c-MYC* and *PKM2*). Some of these associations are the focus of some studies, even if in some cases its function is not fully understood. It is already reported the connection of P53, a transcription factor, with the proteins: YBX1 (P53 is essential for YBX1 nuclear location and YBX1 can affect the P53-regulated transcription) [23]; and c-MYC (this protein can be repressed in a P53-dependent manner) [54]. YBX1 is also linked to *c-MYC* (it can activate the transcription of the *c-MYC* gene) [23]. Also, Cyclin D1 is reported to interact with: FUS (FUS inhibits protein Cyclin D1 expression in human) [17]; YBX1 (suppression of *YBX1* expression decreases the amount of Cyclin D1) [23]; and PKM2 (PKM2 is part of the transcriptional complex for *CCND1* gene expression) [39]. PKM2 is related to: *c-MYC* (similarly to the relation with *CCND1*, is also part of the transcriptional complex for *c-MYC* gene expression) [39]; and PTBP1 (which promotes the expression of *PKM2* by alternative splicing, repressing the expression of *PKM1*) [55]. Furthermore, c-MYC is the transcription factor of *PTBP1* [56]. Assembling this last data, a complex positive feedback-loop occurs between PKM2/c-MYC/PTBP1. Also, our correlation analysis highly supports some of these gene associations (with exception of *FUS*/*CCND1*, *c-MYC/TP53* and *c-MYC/PKM2*), either being direct or indirect interactions. Nevertheless, it is important to highlight that some of these associations occur between the RNA and the protein and for that reason, it would be interesting to evaluate their protein levels to further validate the relation between these gene products in FMCs. Although the evaluation of the proteins in FMC will be interesting, the lack of fresh tumour samples challenges this type of studies. Moreover, most of the works evaluate the protein expression instead of RNA, making difficult to compare our data, but at the same time reinforcing the significance of this work.

The FMC samples here analysed were previously well characterized regarding a considerable set of clinicopathological parameters, making possible to integrate them with the expression data. The parameter tumour size was significantly associated with the expression of *YBX1* and *TP53*. *TP53* overexpression was already reported to be associated with tumour size in HBC [57], as well as, YBX1 [58] at the protein level. However, the *TP53* RNA association with tumour size, in Post hoc Tests, was not significant between size categories, possibly due to the limited number of tumours in some groups, highlighting the need to increase the population to further evaluate this parameter. In parallel, the presence of skin ulceration in cats was found to be associated with *c-MYC*’s expression, and it was already reported that c-MYC plays a role in the inhibition of epithelialization and wound healing [59]. Furthermore, lymph node metastasis was positively associated with *c-MYC* expression; an association also found for *c-MYC* protein levels in HBC patients [32].

Malignancy grade is a helpful tool in HBC and has been suggested as a prognostic biomarker in FMCs [60]. In our analysis when using the EE grading system [42] for the malignancy classification, a relation was found between this parameter and *PKM2* RNA levels, being the sample less malignant, the one that register the highest expression level. However, two of the categories rely on a small number of individuals. In addition, when we classified the malignancy grade by the Mills grading system [43], we did not find any statistically significant result. In the future, it will be important to increase the population studied, specifically with the inclusion of individuals with different tumour grading. Furthermore, our analysis revealed an association between the expression of *PKM2* and the molecular classification of the tumours. The tumours were classified in six molecular subtypes: Luminal A, Luminal B, Luminal B/HER2-negative, HER2-positive, Triple negative basal-like and Triple negative normal-like. Interestingly, an increase in *PKM2* expression was observed in Luminal A tumours and a decrease of this gene expression was found in the Triple negative normal-like tumours, which are associated with better and worse outcomes, respectively [2], suggesting that *PKM2* RNA levels can be used as cancer biomarker. Also, it is important to highlight that *PKM2* expression can be influenced by different signalling pathways, which can be stimulated by the tumour microenvironment (hypoxia and nutrient status), mutations, growth factors (it is described that the PKM2 function and/or transcription is influenced by the signalling of tyrosine kinase receptors as EGFR) and hormones [61], what can be related with our data.

Finally, in our study, the clinicopathological parameter that showed to be preferentially associated with the expression levels was the oral contraceptive administration, being linked with the overexpression of *TP53*, *CCND1*, *FUS*, *YBX1* and *PTBP1*. In fact, the administration of oral contraceptive to domestic animals has been associated with an increased risk in developing tumours, including mammary tumours [62]. Some authors support that over the past forty years, cats have received an excessive dosage of hormones to control reproductive cycles and believe that the administration of lower doses of such compounds and the option for more recent molecules would be potentially safer [63].

**Fig 9:**
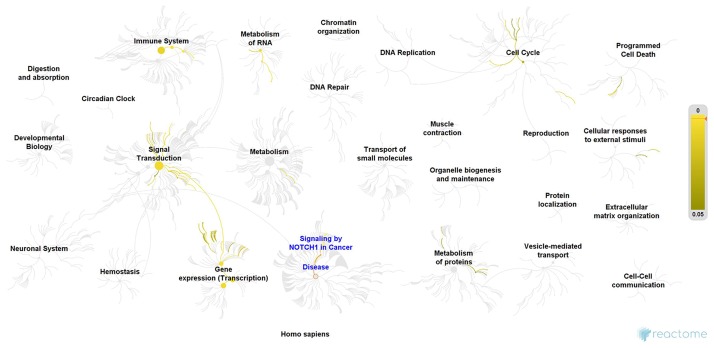
Reactome pathway analysis output. Pathway enrichment analysis of the seven studied genes.

This work demonstrated that many of the cancer-related genes here in analysis are directly associated with each other but may also, indirectly, influence many others, creating a complex molecular cancer network. To further understand this association, we performed a Reactome pathway analysis [64], which revealed that these seven genes are involved in almost 25 interconnected pathways (sum of pathways in which these genes play a role, Fig 9), associated with cell proliferation, apoptosis, cell invasion, gene expression regulation, among others. We found that several of these genes (as *CCND1*, *TP53*, *MYC* and *YBX1*) are involved in the Notch signalling pathway. This pathway is aberrantly activated in breast cancer and have multiple roles during breast tumour progression, including cell proliferation, apoptosis and cancer stem cell activity. Furthermore, elevated Notch signalling has been correlated with therapy resistance in estrogen receptor-positive breast cancer, with the inhibition of Notch receptors and ligands being proposed as a tool to development efficient therapies [65, 66]. These data explain the obtained results regarding the correlation between the expression levels of the genes in study and justifies further research in this issue. Furthermore, our data highlight the similarities between the molecular pathways of HBC and FMCs since the expression data for most of the genes are comparable.

## Conclusions

This work brings new insights in the transcription levels of some cancer-related genes, namely *TP53*, *CCND1*, *FUS*, *YBX1*, *PTBP1*, *c-MYC* and *PKM2* in FMCs following an approach that overcome the germline polymorphisms (since the disease-free tissue from the same animal was used as reference). Some interesting data were obtained regarding the associations found with the clinicopathological parameters. Besides, with this work, was possible to verify that many of these cancer-related genes are correlated but may also, indirectly, influence others genes, creating a complex molecular cancer network. In sum, this type of work, which is focused on the association of cancer-related genes, is essential because it emphasizes the importance of FMCs as a model for HBC research and allows the discovery of putative cancer biomarkers.

## Supporting information

S1 Table Sequence of the primers used in this work. (DOCX)

S2 Table Standard curve parameters. (DOCX)

S3 Table *TP53* RNA quantification of each FMC sample using the DFT sample from the same individual as reference. Values are mean ± SD.
(DOCX)

S4 Table *CCND1* RNA quantification of each FMC sample using the DFT sample from the same individual as reference. Values are mean ± SD.
(DOCX)

S5 Table *FUS* RNA quantification of each FMC sample using the DFT sample from the same individual as reference. Values are mean ± SD.
(DOCX)

S6 Table *YBX1* RNA quantification of each FMC sample using the DFT sample from the same individual as reference. Values are mean ± SD.
(DOCX)

S7 Table *PTBP1* RNA quantification of each FMC sample using the DFT sample from the same individual as reference. Values are mean ± SD.
(DOCX)

S8 Table *c*-*MYC* RNA quantification of each FMC sample using the DFT sample from the same individual as reference. Values are mean ± SD.
(DOCX)

S9 Table *PKM2* RNA quantification of each FMC sample using the DFT sample from the same individual as reference. Values are mean ± SD.
(DOCX)
