# Supplementary material for: Gene expression association study in feline mammary carcinomas
Source: PLoS One. 2019 Aug 28;14(8):e0221776. doi: 10.1371/journal.pone.0221776 (PMC6713336; doi:10.1371/journal.pone.0221776)
Supplement: S1 Table — (DOCX) [file pone.0221776.s001.docx]

**S1 Table.** Sequence of the primers used in this work.

| Gene | Primer Forward Sequence | Primer Reverse Sequence |
| --- | --- | --- |
| *TP53* | TTCACCCTTCAGATCCGTGG | CTTCAGGTGGCTGGAGTGAG |
| *CCND1* | GAACTACCTGGACCGCTTCC | TAGATGCACAGCTTCTCGGC |
| *c-Myc* | AGCAAACCTCCTCACAGCCC | ACTGTCCAACTTGACCCTCT |
| *YBX1* | TGCAGCAGACCGTAACCATT | ACTCTCCGATCCCTCGTTCT |
| *FUS* | AGCAGTGGTGGCTATGAACC | ATGACGTGATCCTTGGTCCC |
| *PTBP1* | CTTCCAGAAGGACCGCAAGA | CCCTAGATGGTGGACTTGGA |
| *PKM2* | TAAGACCCTGTTCGGGTGAG | GCTTCCAGTCCTGCAAACTC |
