# Supplementary material for: Gene expression association study in feline mammary carcinomas
Source: PLoS One. 2019 Aug 28;14(8):e0221776. doi: 10.1371/journal.pone.0221776 (PMC6713336; doi:10.1371/journal.pone.0221776)
Supplement: S2 Table — (DOCX) [file pone.0221776.s002.docx]

**S2 Table.** Standard curve parameters.

| Sequence | r^2^ | Efficiency (%) |
| --- | --- | --- |
| *TP53* RNA | 0.997 | 104.55 |
| *CCND1* RNA | 0.999 | 95.02 |
| *FUS* RNA | 0.998 | 97.26 |
| *YBX1* RNA | 0.999 | 98.92 |
| *PTBP1* RNA | 0.997 | 98.29 |
| *c-Myc* RNA | 0.998 | 97.40 |
| *PKM2* RNA | 0.999 | 94.00 |
