# Supplementary material for: Gene expression association study in feline mammary carcinomas
Source: PLoS One. 2019 Aug 28;14(8):e0221776. doi: 10.1371/journal.pone.0221776 (PMC6713336; doi:10.1371/journal.pone.0221776)
Supplement: S3 Table — Values are mean ± SD. (DOCX) [file pone.0221776.s003.docx]

**S3 Table.** *TP53* RNA quantification of each FMC sample using the DFT sample from the same individual as reference. Values are mean ± SD.

|  | TP53 RNA | |  | TP53 RNA | |  |
| --- | --- | --- | --- | --- | --- | --- |
|  | Disease-free | Carcinoma |  | Disease-free | Carcinoma | |
| 1 | 1.00 (±0.07) | 1.63 (±0.12) | *14* | 1.00 (±0.03) | 2.70 (±0.11) | |
| 2 | 1.00 (±0.02) | 1.32 (±0.04) | *16* | 1.00 (±0.01) | 2.37 (±0.28) | |
| 3 | 1.00 (±0.14) | 1.50 (±3.36x10^-3^) | *17* | 1.00 (±0.03) | 2.81 (±0.03) | |
| 4 | 1.00 (±0.06) | 1.27 (±0.02) | *18* | 1.00 (±0.02) | 2.05 (±0.13) | |
| 5 | 1.00 (±0.16) | 5.41 (±0.10) | *19* | 1.00 (±0.05) | 1.57 (±0.07) | |
| 6 | 1.00 (±0.33) | 1.20 (±0.13) | *20* | 1.00 (±0.06) | 6.76 (±0.21) | |
| 8 | 1.00 (±0.04) | 0.51 (±0.13) | *21* | 1.00 (±0.03) | 0.50 (±0.01) | |
| 9 | 1.00 (±0.01) | 2.98 (±0.23) | *23* | 1.00 (±0.04) | 1.03 (±0.02) | |
| 10 | 1.00 (±0.01) | 1.11 (±0.04) | *24* | 1.00 (±0.01) | 4.49 (±4.77x10^-3^) | |
| 11 | 1.00 (±0.02) | 0.30 (±0.02) | *25* | 1.00 (±0.07) | 1.50 (±0.03) | |
| 12 | 1.00 (±0.04) | 1.11 (±1.57x10^-3^) | *26* | 1.00 (±0.04) | 1.96 (±0.07) | |
| 13 | 1.00 (±0.03) | 1.03 (±0.04) | *27* | 1.00(±0.01) | 1.24 (±0.05) | |
