# Supplementary material for: Gene expression association study in feline mammary carcinomas
Source: PLoS One. 2019 Aug 28;14(8):e0221776. doi: 10.1371/journal.pone.0221776 (PMC6713336; doi:10.1371/journal.pone.0221776)
Supplement: S4 Table — Values are mean ± SD. (DOCX) [file pone.0221776.s004.docx]

**S4 Table.** *CCND1*RNA quantification of each FMC sample using the DFT sample from the same individual as reference. Values are mean ± SD.

|  | CCND1 RNA | | CCND1RNA | | |  |
| --- | --- | --- | --- | --- | --- | --- |
|  | Disease-free | Carcinoma |  | Disease-free | Carcinoma | |
| 1 | 1.00 (±0.14) | 1.69 (±0.04) | *14* | 1.00 (±0.10) | 12.94 (±0.12) | |
| 2 | 1.00 (±0.15) | 4.42 (±0.41) | *16* | 1.00 (±0.13) | 1.54 (±0.49) | |
| 3 | 1.00 (±0.13) | 0.20 (±0.01) | *17* | 1.00 (±0.06) | 2.43 (±0.49) | |
| 4 | 1.00 (±1.00x10^-3^) | 4.19 (±0.33) | *18* | 1.00 (±4.00x10^-3^) | 48.04 (±12.24) | |
| 5 | 1.00 (±0.03) | 48.02 (±2.81) | *19* | 1.00 (±0.03) | 4.14 (±0.21) | |
| 6 | 1.00 (±0.10) | 3.51 (±0.03) | *20* | 1.00 (±0.15) | 157.15 (±15.35) | |
| 7 | 1.00 (±0.02) | 11.95 (±2.76) | *21* | 1.00 (±0.20) | 0.89 (±0.03) | |
| 8 | 1.00 (±0.12) | 1.08 (±0.01) | *23* | 1.00 (±0.07) | 0.82 (±0.03) | |
| 9 | 1.00 (±0.05) | 6.82 (±1.08) | *24* | 1.00 (±0.03) | 0.73 (±0.09) | |
| 10 | 1.00 (±0.12) | 0.36 (±0.01) | *25* | 1.00 (±0.01) | 10.37 (±2.89) | |
| 11 | 1.00 (±0.12) | 0.14 (±2.74x10^-3^) | *26* | 1.00 (±0.02) | 0.71 (±0.05) | |
| 12 | 1.00 (±0.09) | 0.84 (±0.16) | *27* | 1.00(±0.06) | 2.71 (±0.15) | |
| 13 | 1.00 (±0.30) | 1.16 (±0.14) |  |  |  | |
