# Supplementary material for: Gene expression association study in feline mammary carcinomas
Source: PLoS One. 2019 Aug 28;14(8):e0221776. doi: 10.1371/journal.pone.0221776 (PMC6713336; doi:10.1371/journal.pone.0221776)
Supplement: S5 Table — Values are mean ± SD. (DOCX) [file pone.0221776.s005.docx]

**S5 Table.** *c*-*MYC* RNA quantification of each FMC sample using the DFT sample from the same individual as reference. Values are mean ± SD.

|  | *c-MYC* RNA | |  | *c-MYC* RNA | |
| --- | --- | --- | --- | --- | --- |
|  | Disease-free | Carcinoma |  | Disease-free | Carcinoma |
| 1 | 1.00 (±0.04) | 0.05 (±0.01) | *14* | 1.00 (±0.27) | 0.69 (±0.03) |
| 2 | 1.00 (±0.05) | 1.14 (±0.07) | *16* | 1.00 (±0.07) | 0.88 (±0.01) |
| 3 | 1.00 (±0.13) | 0.18 (±0.01) | *17* | 1.00 (±0.24) | 0.74 (±0.10) |
| 4 | 1.00 (±0.03) | 1.22 (±0.07) | *18* | 1.00 (±0.09) | 4.50 (±0.26) |
| 5 | 1.00 (±0.05) | 6.54 (±0.31) | *19* | 1.00 (±0.10) | 1.54 (±0.19) |
| 6 | 1.00 (±0.16) | 3.07 (±0.07) | *20* | 1.00 (±0.04) | 3.37 (±0.12) |
| 7 | 1.00 (±0.09) | 12.01 (±0.18) | *21* | 1.00 (±0.02) | 0.80 (±0.02) |
| 8 | 1.00 (±0.09) | 0.75 (±0.12) | *22* | 1.00 (±0.12) | 7.04 (±1.02) |
| 9 | 1.00 (±0.02) | 1.43 (±0.10) | *23* | 1.00 (±0.07) | 0.73 (±0.14) |
| 10 | 1.00 (±0.12) | 0.51 (±0.06) | *24* | 1.00 (±0.15) | 2.49 (±0.12) |
| 11 | 1.00 (±0.27) | 0.07 (±6.81x10^-3^) | *25* | 1.00 (±0.02) | 1.29 (±0.06) |
| 12 | 1.00 (±0.07) | 1.90 (±0.13) | *26* | 1.00 (±2.73x10^-4^) | 0.98 (±0.14) |
| 13 | 1.00 (±0.03) | 1.30 (±0.32) | *27* | 1.00(±0.23) | 1.18 (±0.02) |
