# Supplementary material for: Gene expression association study in feline mammary carcinomas
Source: PLoS One. 2019 Aug 28;14(8):e0221776. doi: 10.1371/journal.pone.0221776 (PMC6713336; doi:10.1371/journal.pone.0221776)
Supplement: S6 Table — Values are mean ± SD. (DOCX) [file pone.0221776.s006.docx]

**S6 Table.** *PKM2* RNA quantification of each FMC sample using the DFT sample from the same individual as reference. Values are mean ± SD.

|  | *PKM2* RNA | |  | *PKM2* RNA | |
| --- | --- | --- | --- | --- | --- |
|  | Disease-free | Carcinoma |  | Disease-free | Carcinoma |
| 1 | 1.00 (±0.16) | 25.07 (±0.14) | *15* | 1.00 (±0.16) | 99.04 (±15.43) |
| 2 | 1.00 (±0.11) | 0.52 (±0.05) | *16* | 1.00 (±0.02) | 10.13 (±0.85) |
| 3 | 1.00 (±0.09) | 1.52 (±0.09) | *17* | 1.00 (±0.04) | 3.20 (±0.15) |
| 4 | 1.00 (±0.09) | 1.52 (±0.09) | *18* | 1.00 (±0.14) | 29.93 (±4.08) |
| 5 | 1.00 (±0.10) | 3.71 (±0.05) | *19* | 1.00 (±0.03) | 3.90 (±0.24) |
| 6 | 1.00 (±0.27) | 29.16 (±2.98) | *20* | 1.00 (±0.03) | 9.03 (±0.29) |
| 7 | 1.00 (±0.05) | 26.94 (±1.19) | *21* | 1.00 (±0.07) | 0.56 (±1.92x10^-4^) |
| 8 | 1.00 (±0.09) | 2.33 (±0.08) | *22* | 1.00 (±0.07) | 6.15 (±0.18) |
| 9 | 1.00 (±0.03) | 6.58 (±0.85) | *23* | 1.00 (±0.16) | 0.55 (±5.15x10^-4^) |
| 10 | 1.00 (±0.03) | 0.57 (±0.06) | *24* | 1.00 (±0.04) | 1.06 (±0.09) |
| 11 | 1.00 (±0.02) | 0.27 (±0.01) | *25* | 1.00 (±0.01) | 8.74 (±2.57) |
| 12 | 1.00 (±0.07) | 0.74 (±5.22x10^-3^) | *26* | 1.00 (±0.05) | 7.11 (±0.12) |
| 13 | 1.00 (±0.15) | 14.23 (±0.90) | *27* | 1.00(±0.03) | 0.96 (±0.02) |
| 14 | 1.00 (±0.03) | 3.81 (±0.04) |  |  |  |
