# Supplementary material for: Gene expression association study in feline mammary carcinomas
Source: PLoS One. 2019 Aug 28;14(8):e0221776. doi: 10.1371/journal.pone.0221776 (PMC6713336; doi:10.1371/journal.pone.0221776)
Supplement: S7 Table — Values are mean ± SD. (DOCX) [file pone.0221776.s007.docx]

**S7 Table.** *YBX1* RNA quantification of each FMC sample using the DFT sample from the same individual as reference. Values are mean ± SD.

|  | YBX1 RNA | |  | YBX1 RNA | |  |
| --- | --- | --- | --- | --- | --- | --- |
|  | Disease-free | Carcinoma |  | Disease-free | Carcinoma | |
| 1 | 1.00 (±0.10) | 0.63 (±0.05) | *14* | 1.00 (±0.05) | 7.67 (±0.60) | |
| 2 | 1.00 (±0.02) | 1.14 (±0.32) | *16* | 1.00 (±0.08) | 5.21 (±0.24) | |
| 3 | 1.00 (±0.07) | 0.11 (±0.01) | *17* | 1.00 (±0.20) | 0.69 (±0.04) | |
| 4 | 1.00 (±0.06) | 5.23 (±0.20) | *18* | 1.00 (±4.00x10^-3^) | 12.30 (±0.98) | |
| 5 | 1.00 (±0.03) | 6.58 (±0.53) | *19* | 1.00 (±0.12) | 2.13 (±0.06) | |
| 6 | 1.00 (±0.05) | 7.89 (±0.64) | *20* | 1.00 (±2.00x10^-3^) | 10.15 (±0.06) | |
| 7 | 1.00 (±0.01) | 6.93 (±0.05) | *21* | 1.00 (±0.11) | 0.83 (±0.02) | |
| 8 | 1.00 (±0.05) | 0.87 (±0.02) | *23* | 1.00 (±0.08) | 0.49 (±0.03) | |
| 9 | 1.00 (±0.08) | 1.57 (±0.07) | *24* | 1.00 (±0.14) | 1.12 (±0.14) | |
| 10 | 1.00 (±0.22) | 0.27 (±0.05) | *25* | 1.00 (±0.09) | 1.53 (±0.01) | |
| 11 | 1.00 (±0.20) | 0.09 (±2.42x10^-3^) | *26* | 1.00 (±0.07) | 0.94 (±0.16) | |
| 12 | 1.00 (±0.29) | 0.96 (±0.19) | *27* | 1.00(±0.01) | 1.64 (±0.02) | |
| 13 | 1.00 (±0.06) | 2.72 (±0.05) |  |  |  | |
