# Supplementary material for: Gene expression association study in feline mammary carcinomas
Source: PLoS One. 2019 Aug 28;14(8):e0221776. doi: 10.1371/journal.pone.0221776 (PMC6713336; doi:10.1371/journal.pone.0221776)
Supplement: S8 Table — Values are mean ± SD. (DOCX) [file pone.0221776.s008.docx]

**S8 Table.** *FUS* RNA quantification of each FMC sample using the DFT sample from the same individual as reference. Values are mean ± SD.

|  | FUS RNA | |  | FUS RNA | |  |
| --- | --- | --- | --- | --- | --- | --- |
|  | Disease-free | Carcinoma |  | Disease-free | Carcinoma | |
| 1 | 1.00 (±0.08) | 0.25 (±0.03) | *14* | 1.00 (±0.11) | 3.13 (±0.20) | |
| 2 | 1.00 (±0.09) | 1.53 (±0.24) | *16* | 1.00 (±0.10) | 3.23 (±0.71) | |
| 3 | 1.00 (±0.13) | 0.16 (±0.01) | *17* | 1.00 (±0.10) | 1.68 (±0.03) | |
| 4 | 1.00 (±0.33) | 5.02 (±1.22) | *18* | 1.00 (±0.03) | 12.14 (±1.15) | |
| 5 | 1.00 (±0.12) | 21.41 (±0.90) | *19* | 1.00 (±0.09) | 1.95 (±0.21) | |
| 6 | 1.00 (±0.11) | 6.02 (±0.48) | *20* | 1.00 (±0.04) | 6.91 (±0.86) | |
| 8 | 1.00 (±0.01) | 0.41 (±0.02) | *21* | 1.00 (±0.03) | 1.33 (±0.01) | |
| 9 | 1.00 (±0.12) | 3.67 (±0.29) | *23* | 1.00 (±0.13) | 0.74 (±0.07) | |
| 10 | 1.00 (±0.01) | 0.43 (±0.04) | *24* | 1.00 (±0.06) | 0.84 (±0.01) | |
| 11 | 1.00 (±0.02) | 0.06 (±3.88x10^-3^) | *25* | 1.00 (±0.08) | 1.30 (±0.01) | |
| 12 | 1.00 (±0.19) | 0.85 (±0.09) | *26* | 1.00 (±0.13) | 0.99 (±0.11) | |
| 13 | 1.00 (±0.12) | 1.99 (±0.25) | *27* | 1.00(±0.06) | 1.47 (±4.14x10^-3^) | |
