# Supplementary material for: Gene expression association study in feline mammary carcinomas
Source: PLoS One. 2019 Aug 28;14(8):e0221776. doi: 10.1371/journal.pone.0221776 (PMC6713336; doi:10.1371/journal.pone.0221776)
Supplement: S9 Table — Values are mean ± SD. (DOCX) [file pone.0221776.s009.docx]

**S9 Table.** *PTBP1* RNA quantification of each FMC sample using the DFT sample from the same individual as reference. Values are mean ± SD.

|  | PTBP1 RNA | |  | PTBP1 RNA | | |  |
| --- | --- | --- | --- | --- | --- | --- | --- |
|  | Disease-free | Carcinoma |  | | Disease-free | Carcinoma | |
| 1 | 1.00 (±0.08) | 0.15 (±1.70x10^-3^) | *14* | | 1.00 (±0.11) | 4.61 (±0.52) | |
| 2 | 1.00 (±0.29) | 2.69 (±0.71) | *16* | | 1.00 (±0.06) | 2.66 (±0.35) | |
| 3 | 1.00 (±0.09) | 0.09 (±0.01) | *17* | | 1.00 (±0.07) | 1.70 (±0.38) | |
| 4 | 1.00 (±0.09) | 4.99 (±0.89) | *18* | | 1.00 (±2.00x10^-3^) | 9.87 (±0.86) | |
| 5 | 1.00 (±2.00x10^-3^) | 12.31 (±0.52) | *19* | | 1.00 (±0.11) | 2.19 (±0.28) | |
| 6 | 1.00 (±0.09) | 1.20 (±0.04) | *20* | | 1.00 (±0.02) | 11.19 (±0.04) | |
| 8 | 1.00 (±0.04) | 0.35 (±9.21x10^-4^) | *21* | | 1.00 (±0.19) | 0.89 (±0.03) | |
| 9 | 1.00 (±0.29) | 3.18 (±0.15) | *23* | | 1.00 (±0.07) | 0.53 (±0.08) | |
| 10 | 1.00 (±0.04) | 0.43 (±0.10) | *24* | | 1.00 (±0.07) | 1.17 (±0.15) | |
| 11 | 1.00 (±0.06) | 0.04 (±1.93x10^-3^) | *25* | | 1.00 (±0.03) | 1.88 (±0.01) | |
| 12 | 1.00 (±0.12) | 0.86 (±0.01) | *26* | | 1.00 (±0.03) | 0.70 (±0.10) | |
| 13 | 1.00 (±0.25) | 2.24 (±0.05) | *27* | | 1.00(±0.01) | 1.28 (±0.02) | |
